# Can the robot “see” what I see? Robot gaze drives attention depending on mental state attribution

**DOI:** 10.3389/fpsyg.2023.1215771

**Published:** 2023-07-13

**Authors:** Lucas Morillo-Mendez, Rebecca Stower, Alex Sleat, Tim Schreiter, Iolanda Leite, Oscar Martinez Mozos, Martien G. S. Schrooten

**Affiliations:** ^1^Centre for Applied Autonomous Sensor Systems, Örebro University, Örebro, Sweden; ^2^Division of Robotics, Perception and Learning, KTH, Stockholm, Sweden; ^3^School of Behavioral, Social, and Legal Sciences, Örebro University, Örebro, Sweden

**Keywords:** gaze following, cueing effect, attention, mentalizing, intentional stance, social robots

## Abstract

Mentalizing, where humans infer the mental states of others, facilitates understanding and interaction in social situations. Humans also tend to adopt mentalizing strategies when interacting with robotic agents. There is an ongoing debate about how inferred mental states affect gaze following, a key component of joint attention. Although the gaze from a robot induces gaze following, the impact of mental state attribution on robotic gaze following remains unclear. To address this question, we asked forty-nine young adults to perform a gaze cueing task during which mental state attribution was manipulated as follows. Participants sat facing a robot that turned its head to the screen at its left or right. Their task was to respond to targets that appeared either at the screen the robot gazed at or at the other screen. At the baseline, the robot was positioned so that participants would perceive it as being able to see the screens. We expected faster response times to targets at the screen the robot gazed at than targets at the non-gazed screen (i.e., gaze cueing effect). In the experimental condition, the robot's line of sight was occluded by a physical barrier such that participants would perceive it as unable to see the screens. Our results revealed gaze cueing effects in both conditions although the effect was reduced in the occluded condition compared to the baseline. These results add to the expanding fields of social cognition and human-robot interaction by suggesting that mentalizing has an impact on robotic gaze following.

## 1. Introduction

Most adults and older children possess the cognitive capacity to infer the mental states of others. This capacity is also known as mentalizing, or theory of mind (Premack and Woodruff, 1978; Baron-Cohen, 1995), and functions as a tool to infer the emotions, thoughts, preferences, and intentions of others. Without mentalizing, achieving mutual understanding in human communication would be difficult. We can infer others' internal states through verbal communication, facial expressions, gestures, and gaze. Through gaze, we can perceive and provide information to others (Cañigueral and Hamilton, 2019). This dual function of gaze allows us to look at a point in space and convey a message to a partner so they, too, look at the same point, either reflexively or–as has become clear recently–more strategically, depending on several social factors (Dalmaso et al., 2020). This action would initiate a joint attention process (Tomasello, 1995), a precursor and facilitator of the attribution of mental states (Perez-Osorio et al., 2021).

Gaze following can be investigated using gaze cueing tasks (Friesen and Kingstone, 1998; Driver et al., 1999), a behavioral attention measure based on Posner's spatial cueing task (Posner, 1980). In a typical gaze cueing task, a central cue conveying gaze (usually a pair of eyes) is presented at the center of a screen. This is followed by a change in gaze direction toward the left or right and the appearance of a target stimulus at either of these two locations. The participant's task is to respond to the target's identity or location by pressing one of two response keys. Response times are typically faster for targets appearing at the gazed-at location (valid or congruent trials) than those appearing at the opposite side (invalid or incongruent trials). This phenomenon is known as the gaze cueing effect (Frischen et al., 2007) and reveals the role of gaze in guiding visual attention.

Gaze cues have shown unique results related to social orienting (Frischen et al., 2007). For example, a face showing an emotional expression is associated with a larger cueing effect (McKay et al., 2021). Similarly, the ethnicity of the facial cue also affects the cueing effect (Zhang et al., 2021). Moreover, the decline in eye gaze following is associated with aging (McKay et al., 2022; Morillo-Mendez et al., 2022) and, most relevant for the current study, the gaze cueing effect is moderated by mentalizing (Dalmaso et al., 2020). This moderation is unique to face and gaze cues but not seen with non-social cues such as arrows in similar spatial cueing tasks (Kawai, 2011).

### 1.1. Gaze following and mental state attribution

The role of mental state attribution in gaze following is a current topic of debate. Mental state attribution has been explored by manipulating the occlusion of the line of vision between the cue and the target in gaze cueing tasks by closing the eyes, covering the eye region, or adding a physical barrier (Dalmaso et al., 2020). If mental state attribution has a role in gaze cueing, when the gaze of the other is perceived as non-purposeful–i.e., the cue is perceived as not being able to see the reference object–the gaze cueing effect is expected to be attenuated or even absent. The question is how helpful a gaze cue is if it is not perceived as linked to the reference object. Research is mixed and has shown weaker (Teufel et al., 2010; Schulz et al., 2014), absent (Nuku and Bekkering, 2008; Kawai, 2011), or similar (Cole et al., 2015; Kingstone et al., 2019) effects between conditions where the central cue is or is not perceived as being able to see the target.

The schema theory of gaze cueing proposed by Cole et al. (2015) suggests that gaze following is triggered automatically, as a learned schema of joint attention, that cannot be easily suppressed (Cooper and Shallice, 2000). Whether or not the schema is triggered can vary depending on the strength of the learned schema or mental state attribution, i.e., top-down influences. This theory predicts that in situations where the gaze direction is unambiguous, a powerful influence would be needed to suppress the activated schema, as found in research using real pictures/videos of humans or physically present humans with uncovered eyes (Cole et al., 2015; Kingstone et al., 2019). In situations where the gaze cue is more ambiguous, such as when the eyes are occluded, the activation of the schema would be more easily modulated, for instance, by having the idea that the gaze cue can (or cannot) see the target (Nuku and Bekkering, 2008; Teufel et al., 2010; Kawai, 2011; Schulz et al., 2014).

To address the need for ecologically valid research in attention with complex stimuli and the increasing use of social robots in everyday settings, we developed a situated gaze cueing task using a NAO robot (Gouaillier et al., 2009) to investigate the role of mental state attribution in robotic gaze following. While recent, the use of robots to study gaze following and joint attention is not new (see Chevalier et al., 2019 for a review).

### 1.2. Robots, gaze following, and the adoption of the intentional stance

The use of artificial embodied agents in experimental research permits causal inferences to be derived from human-robot interactions (HRIs), which has specific implications. First, this approach adds to a better understanding of social cognition in response to artificial stimuli (Wykowska, 2020). Second, this approach can also inform the computational cognitive models of robots designed to assist humans, ultimately increasing their effectiveness in social communication (Cross et al., 2019; Wykowska, 2021). Finally, robots in face-to-face experiments are more sophisticated stimuli than two-dimensional screen-based images while still permitting highly controlled experimentation (Wykowska, 2020).

Gaze cueing effects of robotic gaze have been consistently found with different anthropomorphic robots, including sophisticated ones with eye movement (Kompatsiari et al., 2021; Ciardo and Wykowska, 2022) and those without eye movement (Chaminade and Okka, 2013; Morillo-Mendez et al., 2022, 2023). This finding does not automatically categorize such robots as social agents as non-social stimuli such as arrows can also induce automatic gaze cueing effects (Kawai, 2011; Slessor et al., 2016). However, anthropomorphic robots can be perceived as mindful through appearance alone (Martini et al., 2016). In this line, Morillo-Mendez et al. (2023) showed that the gaze direction of a NAO robot, not the mere motion of its head movement, reflexively drove visual attention. The gaze cueing effect did not depend on whether the robot was presented frontally, with its eyes fully visible to the participants or faced away from the participants, with its eyes not visible. Therefore, when the robot faced away, the gaze cueing effect persisted despite the absence of direct eye cues and the presence of an opposing motion cue (compared to when the participants saw the robot's face from the front). Therefore, it is logical to cautiously infer that perspective-taking played a role in the appearance of this gaze cueing effect.

The role of mental state attribution in robotic gaze following has not yet been directly addressed. However, previous studies have explored the role of the so-called intentional stance in joint attention with a robotic agent in gaze cueing tasks with robots (Abubshait and Wykowska, 2020; Willemse et al., 2022). The adoption of the intentional stance (Dennett, 1989) refers to the adoption of the strategy in explaining the behavior of artifacts, whether they are cartoons, virtual agents, schematic faces, or robots, in terms of having mental states. Mentalizing refers to inferring these mental states in a specific context (Perez-Osorio et al., 2021). Notably, adopting the intentional stance and mentalizing are closely related, with adopting the intentional stance possibly being required to make mental state attributions and vice versa.

Humans adopt the intentional stance to some extent in interactions with robots (Thellman et al., 2017; Marchesi et al., 2019). Moreover, research has shown that the lack of adoption of the intentional stance, induced through instructions from experimenters, can significantly reduce the gaze cueing effect with robotic and human cues (Wykowska et al., 2014).

We designed a gaze cueing task with a NAO robot to specifically explore the role of mental state attribution in gaze following with a robotic agent. We used a NAO robot given its popularity and wide use (Gelin, 2017). Potential differences in the gaze cueing effect between conditions in which the robot “can” or “cannot” see the target would imply a role of mental state attribution in the gaze cueing effect. The main research question of this study is whether mental state attribution affects robotic gaze following.

## 2. Methods

The methods of this study were pre-registered before data collection at the Open Science Framework (OSF) (https://doi.org/10.17605/OSF.IO/ZTQ9G).

### 2.1. Participants

The inclusion criteria to participate were to be fluent in English, to have a normal or corrected-to-normal vision, and to be at least 18 years old. Participation was voluntary, based on informed consent, and in exchange for a 100 SEK gift voucher. Forty-nine volunteers participated in the experiment. Data from one participant was corrupted due to a robot issue and excluded from the analysis. Two additional participants with an excessive number of errors were also excluded (see subsection 2.5). This resulted in a final sample of 46 participants (mean age = 28 ± 5.45 years; 18 women, 28 men, one left-handed). Participants reported being very comfortable using computers (mean score = 4.7 ± 0.75 out of 5), being familiar with NAO (63% had seen it before), and most of them already participated in previous research with a robot in the past (also 63%). They also reported a mean of 17.5 ± 2.7 years of education.

We calculated the minimum sample size before starting the data collection by performing a power analysis using G-Power (Faul et al., 2007) with $\eta_{p}^{2}$ = .08 and 1 − β = 0.8. The analysis returned a minimum number of 34 participants as sufficient for our design, fewer than our final sample.

### 2.2. Experimental design and measures

The experimental design consisted of a within-subject manipulation of two independent variables: (a) the congruence of robotic eye gaze and target location (two levels: congruent vs. incongruent) and (b) the occlusion of the line of sight between the robot and the screen (two levels: baseline vs. occluded). The dependent variables were accuracy and reaction time (RT), defined as the time between the onset of the target and the participant's response.

**Figure 1:**
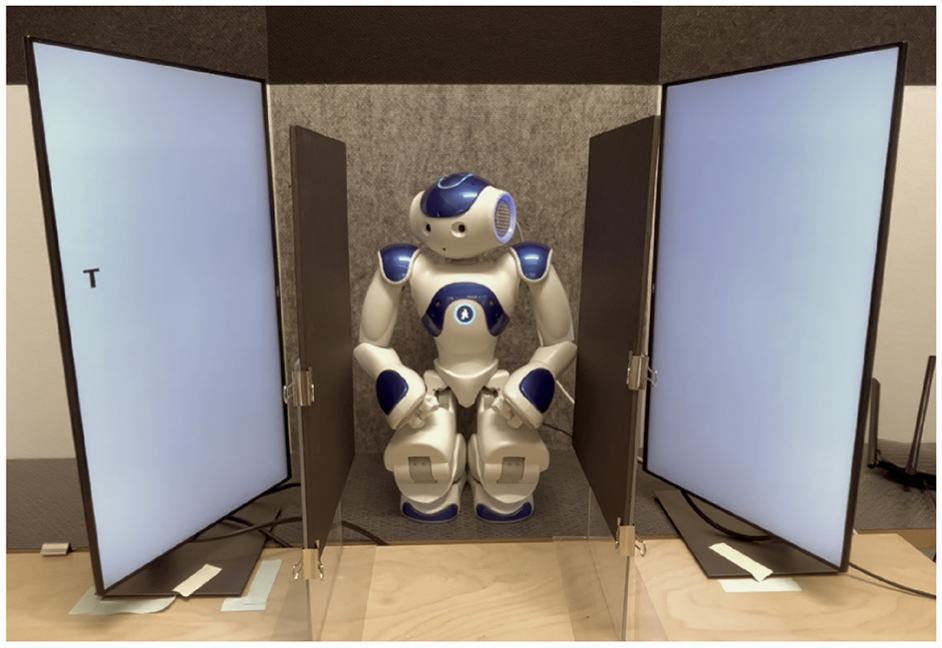
Settings from the participant's perspective in the occluded condition.

We hypothesized that responses to the target would be faster on congruent trials than on incongruent trials (i.e., gaze cueing effect) in the baseline condition, in which the line of sight of the robot is clear. A reduced gaze cueing effect when the robot-target line of sight is occluded (Figure 1), would suggest that mental attribution modulates the gaze cueing effect present in the baseline (Dalmaso et al., 2020). This would be reflected in a congruence × occlusion interaction.

Participants filled out a brief demographic questionnaire in which they reported their gender [man, woman, other, and N/A], age in years, dominant hand, comfort with computers [Likert scale, 1–5], years of education, familiarity with the NAO robot [yes, no, and not sure], and previous participation in studies involving robots [yes, no, and not sure].

### 2.3. Task design, apparatus, and stimuli

The participants performed a gaze cueing task in an environment with a NAO robot and two vertically aligned screens placed by its sides. Instead of using a central fixation cross, typical in screen-based studies, the robot's eyes flashed to attract the participant's attention and eye focus. Then, the head of the NAO turned either to its left or right, immediately followed by a target letter (“V” or “T”) which appeared on either the left or right screen at the robot's eye level. In half of the trials, the target location was congruent with the direction of the robotic eye gaze, while in the other half, the target location and the eye gaze direction were incongruent. The participants had to identify the target letter by pressing one of two response keys as quickly as possible while minimizing mistakes. During the task, eye movements were recorded with a Tobii Pro Glasses 3. The analysis of eye-tracking data is beyond the scope of this article and will be published separately.

**Figure 2:**
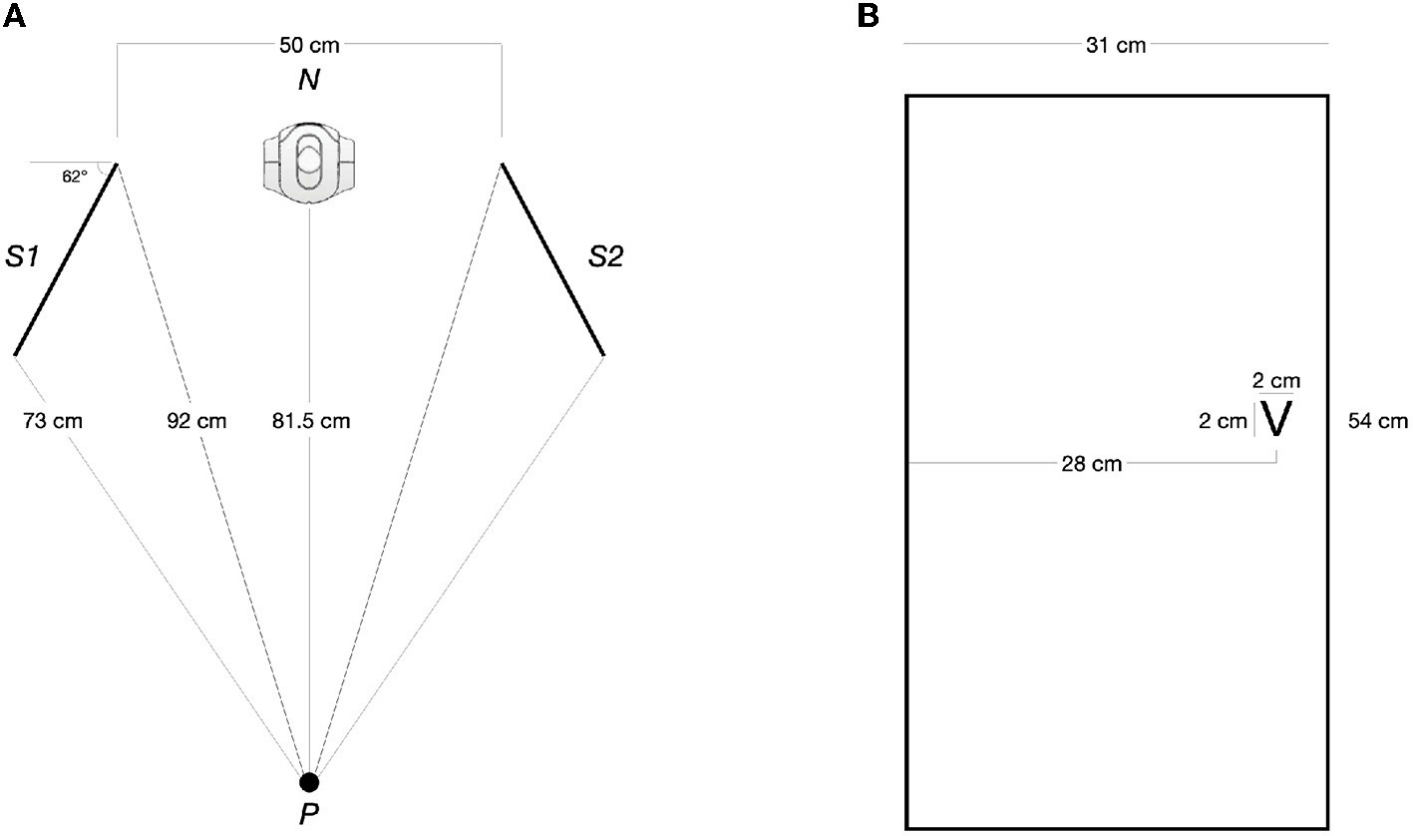
**(A)** Zenithal view with the measures of the main elements in the scenario; Participant (P) faces the NAO robot (N), with two screens (S1 and S2) to its right and left, respectively. **(B)** Dimension and placement of the target letter within a screen. The image corresponds to S2 - the letter's position mirrors S1.

Stimulus presentation and response registration were done using the PsychoPy library for Python (Peirce et al., 2019). The target letters were presented on two identical 24-inches Dell monitor screens with a refresh rate of 60 Hz (1,920 x 1,080 pixels). Participants used a *Cedrus* response keypad RB-540. The mapping of the hands-on response keys was counterbalanced between participants; that is, half were instructed to respond with their left index finger to the “T” (upper key) and with their right index finger to the ‘V' (lower key); the other half used the opposite hand-key mapping. The movement of the robot head consisted of a head yaw movement of 35° toward its left or right. Other measures and distances of the setting can be seen in Figure 2.

**Figure 3:**
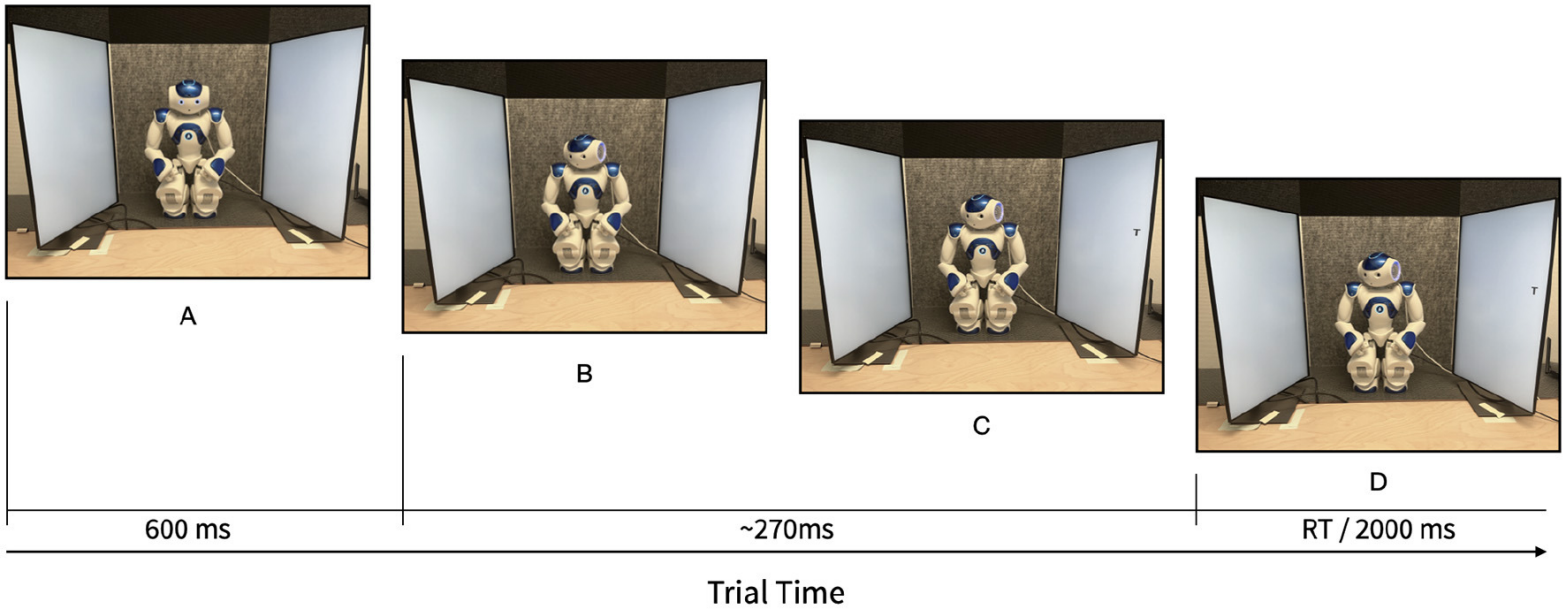
Trial times. **(A)** Flashing eyes (fixation signal). **(B)** Head rotation (complete duration~220 ms). **(C)** Target onset. **(B, C)** Time between onset of head movement and target onset, or stimulus onset asynchrony (SOA). **(D)** Time until response.

The gaze cueing task consisted of 10 blocks of 26 trials each. Congruence was manipulated trial-by-trial, while occlusion was manipulated between blocks. The first two trials of each block were random and excluded from the analysis. The remaining experimental trials within each block contained an equal number of trials for each combination of letter identity, gaze direction, and congruence between eye gaze direction and target location. This configuration resulted in eight unique types of trials and 24 trials per block as potential candidates for analysis, so every type of trial was presented three times per block. Figure 3 shows an example of a full trial, with a stable stimulus onset asynchrony (SOA) of 270 ms between the onset of the head movement and the appearance of the target stimulus. Previous literature has shown gaze cueing effects with SOAs starting at 105 ms (Friesen and Kingstone, 1998; Chevalier et al., 2019). With robot cues, gaze cueing effects have been reported at SOAs as between 300 ms (Chaminade and Okka, 2013) and 1,000 ms (Kompatsiari et al., 2018; Morillo-Mendez et al., 2023). Within each block, the experimental trials were presented in a different random order for each participant, with the constraint that the same trial type could not appear more than two times consecutively.^1^ The order of the blocks was counterbalanced between participants, with half starting with five occluded blocks and half with five baseline blocks.^2^ A Wilcoxon rank sum test showed no differences in RT between the two block orders, *p* = .06.

### 2.4. Procedure

The experiment took place in a quiet room in the KTH division of Robotics, Perception, and Learning. Upon arrival, participants gave written consent after being informed about the aim of the study, the use of their data, and their rights, as well as the possibility of terminating participation at any stage without any risk of penalty. Additionally, participants filled out the demographic questionnaire.

During the instruction phase, participants were informed that the robot's gaze would not predict the letter's location. They were encouraged to minimize their mistakes while responding as quickly as possible. Accuracy and mean reaction time appeared at the end of each block to maintain the participant's motivation during the task. Additionally, they were instructed that at the beginning of a trial, they should look at the eyes of the robot when they flashed. Compliance with these instructions was monitored during the following 10 practice (baseline) trials using the real-time video feed from the eye-tracking glasses. After the training, the instructions were repeated, and any questions or clarifications from the participants were addressed.

All participants wore noise-canceling headphones during the task. If compliance with the instruction was compromised during task performance, participants were reminded of the instruction in the next immediate break between blocks. Participants took self-paced breaks between blocks. The experimenter entered the room between different conditions to remove or add the occluding barriers and reminded the participant that the instructions remained the same. The complete session lasted 45 min. Upon completion, participants were debriefed about the experiment's aim in detail, received a gift voucher, and were thanked for participating.

### 2.5. Statistical analysis

Before the analysis, we identified participants with an unusually high number of errors compared to the rest of the sample and excluded them as outliers. Errors were defined as trials in which the reported letter was incorrect, the number of responses was higher than one, or 2 s had passed without a response from the participant. Two participants exhibited an extreme number of errors (19% and 45% of the trials; >*Q*3+3*IQR) and were therefore excluded from the analysis. We excluded the remaining incorrect trials (3.3%) from the RT analysis. Additionally, we removed trials with extreme RT outliers for each participant.

**Figure 4:**
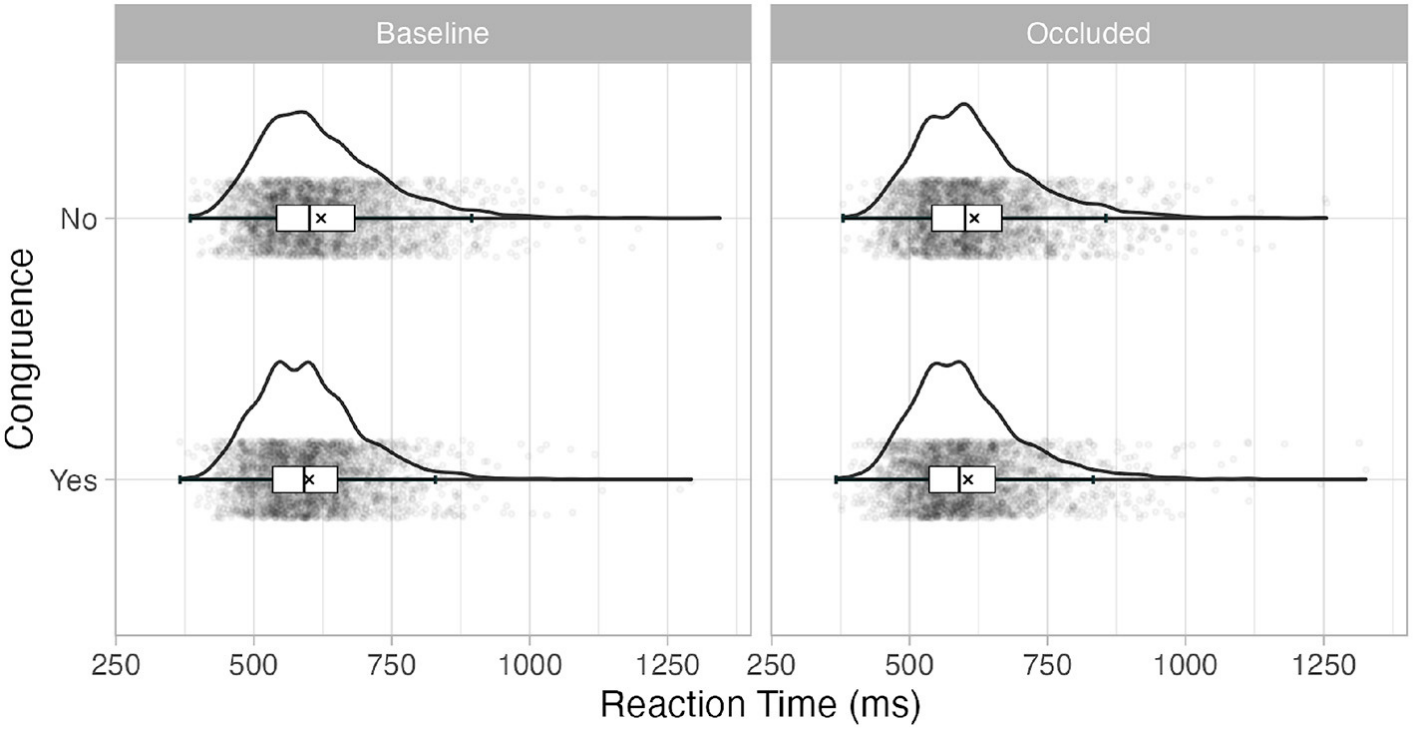
Boxplot and distributions of the raw reaction time at each level of the variables. The ‘×' marks the mean.

We used Generalized Linear Mixed Models (GLMMs) (Breslow and Clayton, 1993) to analyze the RT data as recommended by Lo and Andrews (2015). The GLMM approach accounts for the non-normal distribution of RTs at each variable level combination without the need for data transformation. While data transformation allows the data to satisfy the normality assumptions typically required for statistics based on linear models (including ANOVA), the distortion of the dependent variable may obscure the potential theoretical implications in the results (Lo and Andrews, 2015; Bono et al., 2021). Moreover, (G)LMMs can address the random effects that arise from within-subject designs such as the current (Meteyard and Davies, 2020). The non-normal, positively skewed distribution of RTs in this study can be seen in Figure 4 for each combination of the variable levels. We used an inverse Gaussian distribution with identity link to analyze positively skewed data, following the recommendations of Lo and Andrews (2015).

The analysis was performed in *RStudio* 1.4.1564 (Posit team, 2022) using the *lme4* package (Bates et al., 2015). The model was built using forward selection from a simple model with intercept and random intercept per participant to a final one including those variables of interest that improved the previous model after its addition. The models were estimated with a function of *Maximum Likelihood*, and -2 * log likelihood was used as the goodness-of-fit method. We used chi-square tests to compare the fit between the consecutive models.

## 3. Results

Given our within-subject design, the first model consisted of an intercept and random intercept for participants. The random intercept for participants showed a standard deviation of 23 ms in the final model. We also considered the target letters as a random factor, and this second model showed a significant variance in intercepts across target letters, SD = 1.74, χ^2^(1) = 4.55, *p* = .03.

**Figure 5:**
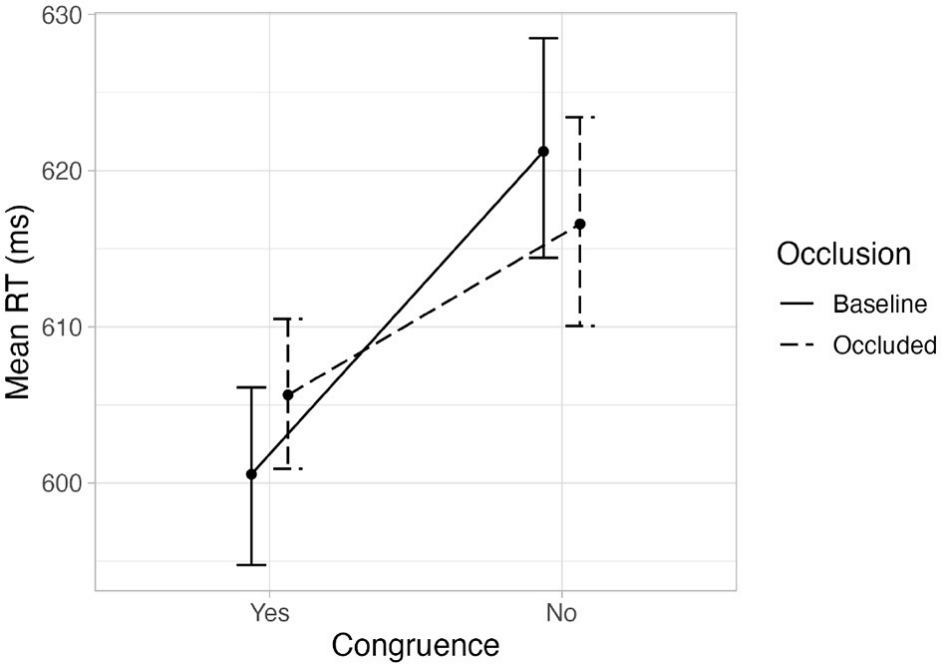
Mean of the aggregated mean reaction time per participant at each level of the variables. The error bars show bootstrapped within-subjects CIs (95%, b = 1,000).

The fixed variables of interest in our experiment were gaze-target congruence, occlusion, and the interaction between them. These were added to the model in this order. Gaze-target congruence improved the model fit significantly when added, χ^2^(1) = 79.36, *p* < .001, but occlusion did not significantly improve the model in which gaze-target congruence was included, χ^2^(1) = 0.26, *p* = .6. Finally, adding the interaction between gaze-target congruence and occlusion significantly improved the model fit, χ^2^(2) = 7.48, *p* = .02. Table 1 shows the final model and its coefficients. Figure 4 shows a violin plot with the distributions of the variables of interest. A graph with the means of the aggregated mean RT per variable and participant can be seen in Figure 5 for comparison purposes with previous research using ANOVA techniques. The corresponding repeated measures 2 x 2 ANOVA also showed a main effect of congruence, *F*(1, 45) = 23.4, *p* < .001, and an interaction effect between congruence and occlusion, *F*(1, 45) = 4.25, *p* = .045.

**Table 1:** Unstandarized slopes of the fixed effects for the final GLMM.

**Fixed effects**	***RT***~***Congruence***+***Congruence***:***Occlusion***+ 1|***Letter***+1|***ID***			
		**b**	**St. Error**	***t*-value**	***p*-value**
Intercept	621.54	8.95	69.4	< .001
Congruence (No)	17.65	2.1	8.43	< .001*
Congruence (Yes): Occluded (Yes)	4.66	2.05	2.26	.02
Congruence (No): Occluded (Yes)	-3.51	2.15	-1.63	.1

*One-tailed (original also < .001).

To explore the gaze-target congruence × occlusion interaction, we built two more GLMMs separated by occlusion condition (baseline and occluded; see Table 2). The analyses showed a significant main effect of gaze-target congruence of robotic eye gaze and target location on RT for each condition. However, this effect seemed larger in the baseline condition. The main effect of congruence remained significant in both models when correcting for multiple comparisons (Holm-Bonferroni method).

**Table 2:** Unstandarized slopes of the fixed effects for the GLMMs segregated by occlusion.

**Occluded = No (Baseline)**	***RT***~***Congruence***+1|***Letter***+1|***ID***			
**Fixed effects**	**b**	**St. Error**	**t-value**	***p*** **-value**
Intercept	621.22	13.67	45.44	< .001
Cong. (No)	17.42	2.08	8.37	< .001
**Occluded** = **Yes**	*RT*~*Congruence*+1|*Letter*+1|*ID*			
**Fixed effects**	**b**	**St. Error**	**t-value**	***p*** **-value**
Intercept	624.43	11.37	54.9	< .001
Cong. (No)	8.9	2.1	4.2	< .001

**Figure 6:**
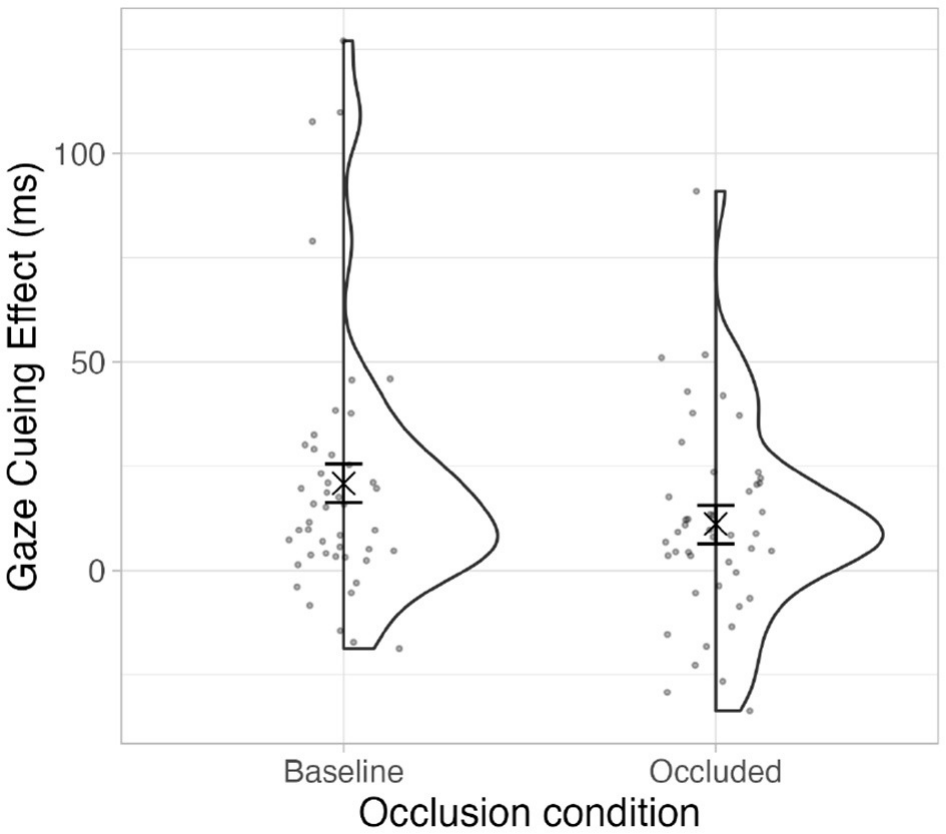
Mean magnitude of the gaze cueing effect for each occlusion condition. The ‘×' marks the mean. The error bars show bootstrapped within-subjects CIs (95%, *b* = 1,000).

The difference in magnitude between the gaze cueing effects in both conditions was calculated as the differences of the aggregated mean values of incongruent and congruent trials per participant and occlusion group. The results of a Wilcoxon signed-rank test showed that the gaze cueing effect was significantly higher in the baseline condition (*Median* = 13*ms*) compared to the occluded condition (*Median* = 9*ms*), V = 739, *p* = .03, *r* = 0.32, Bootstrapped within-subjects CI (95%, b = 1,000) [0.9, 19.92]. The mean values and distribution are in Figure 6.

## 4. Discussion

The current experiment contributes to the growing body of research that suggests that robot behavior is interpreted as mindful and social (Clark and Fischer, 2023; Doyle and Hodges, 2023). We used a gaze cueing task during which a robotic agent could “see” the target or not due to a physical barrier to explore the role of mentalizing in robotic gaze following. Our results revealed gaze cueing effects in both conditions although the effect was significantly reduced in the occluded condition compared to the baseline condition. These results suggest that attentional orienting to robotic gaze cues depends on whether the anthropomorphic robot is perceived as seeing the reference object, which reflects mental state attribution. We will discuss these results here with reference to social cognition and emphasize the implications for HRI research.

Our finding of a reduced gaze cueing effect in the occluded condition compared to the baseline conditions aligns with the schema theory of gaze cueing (Cole et al., 2015). First, the gaze cueing effect at the baseline can be explained by the activation of a joint attention schema based on previous experience. In contrast, the reduced gaze cueing effect in the experimental condition can be taken to reflect a top-down modulation based on context - i.e., “the robot can/cannot see the target”. To further explore the role of top-down modulation, future attempts are needed to replicate these results by including a systematic variation of different SOAs as part of the experiment. SOAs longer than 300 ms can better capture strategic processing (Dalmaso et al., 2020), so the reduction (or elimination) of the gaze cueing effect only in the occluded condition at longer SOAs would emphasize the role of mentalizing as a modulator of joint attention when participants are given more time to (strategically) process the signal.

Second, we used a NAO robot whose gazing capabilities can only be conveyed through head movement. Although effective in inducing consistent cueing effects (Morillo-Mendez et al., 2022, 2023), this rigidity can still be perceived as an ambiguous social cue by the viewer since humans convey gaze through a combination of head and eye movements. According to the schema theory of gaze cueing, mental state attribution would exert more influence in attenuating the gaze cueing effect with more ambiguous gaze cues, such as gaze direction induced by the head's orientation (but not the eyes) of a robot, as in the current experimental setup. Future research might explore the role of mental state attribution in gaze following with other robotic agents, especially those with eye movement.

Our findings indicate a reduction of the cueing effect in the occluded condition rather than a complete absence. However, attenuated gaze cueing effects that cannot be entirely suppressed have been associated with dynamic eye-motion cues (Teufel et al., 2010; Schulz et al., 2014) rather than with static eye-gaze (Nuku and Bekkering, 2008; Kawai, 2011) such as in the present experiment. In a similar vein, the schema theory of gaze cueing states that the cueing effect can be inhibited to a certain extent, and mental state attribution could inhibit the effect when less clear sensory information is available. Since our robot cannot move the eyes, these results are unlikely to be related to an automatic mechanism activated by eye motion (Baron-Cohen, 1995; Schulz et al., 2014). Further research on robotic gaze following should explore social orienting by including robots with varying gazing capabilities.

Some limitations must be acknowledged when interpreting the current, novel findings. First, our sample was biased toward individuals with high familiarity with NAO and HRI experiments and a high level of comfort with computers. It has been suggested that people with technical backgrounds exposed to social robots are more likely to adopt the intentional stance toward a humanoid robot (Roselli et al., 2023). While our main aim was to explore the role of mental state attribution in robotic gaze following, which might be related to intentional stance adoption, an avenue for future studies is to replicate these findings in samples with different predispositions to adopt the intentional stance to better link it with the role of theory of mind in gaze following. Moreover, future research could also address individual differences in the mentalizing capabilities of different groups of the population (Apperly, 2012).

Indeed, research with robotic agents and people with difficulties related to social cognition has shown mixed results, with some studies showing similar gaze following in robots and humans (e.g., as a result of aging, Morillo-Mendez et al. 2022) and others showing different patterns between them (e.g., autism spectrum, Wiese et al. 2014). Given this complex research landscape, future research is warranted to continue exploring social cognition with different models of social robots in diverse populations.

In addition, we used a behavioral measure design and did not use self-reported questionnaires. Future studies should consider the use of questionnaires such as the InStance questionnaire (Marchesi et al., 2019) to explore individual differences in the adoption of the intentional stance and tailored to the specific type of robot used (Metta et al., 2008), a robot with eye movement. Finally, we used a highly controlled experimental paradigm to explore a primary aspect of social cognition. While this paradigm aims to provide an ecologically valid scenario, using a situated robot and involving motion cues, future research should explore open tasks and scenarios with other real robots to see the potential real-world impact of controlled research findings in everyday HRIs.

In conclusion, this study suggests that robotic gaze following depends on mental state attribution, supporting the view that humans adopt the intentional stance toward robots. Our findings contribute to the growing body of research in social cognition and HRI, providing insights into the mechanism underlying gaze following when gaze originates in robotic agents. Further interdisciplinary collaboration between cognitive scientists, psychologists, roboticists, and computer scientists is critical to ensure social agents' evidence-based and user-centered design and their computational behavior models, which would promote the increasing acceptance of robotic social agents.

## Data availability statement

The datasets presented in this study can be found in online repositories. The names of the repository/repositories and accession number(s) can be found below: https://osf.io/jpnzt/.

## Ethics statement

The studies involving human participants were reviewed and approved by Swedish Ethical Review Authority. The patients/participants provided their written informed consent to participate in this study.

## Author contributions

LM-M, RS, IL, OM, and MS contributed to the conception and design of the study. AS and TS coded the task and synchronized the robot's behavior with the response registration. LM-M collected and analyzed the data and wrote the manuscript. All authors reviewed and approved the submitted version.

## Footnotes

^1^Due to a technical error, the trials for the first seven participants were randomized within the whole session instead of within blocks. The analyses were performed with and without these participants and yielded essentially identical results. The reported results and open dataset include the data from these participants (IDs 1–7).

^2^Implemented at https://github.com/alexsleat/naoposner.
